# Idiopathic pulmonary fibrosis and the role of genetics in the era of precision medicine

**DOI:** 10.3389/fmed.2023.1152211

**Published:** 2023-04-27

**Authors:** Aitana Alonso-Gonzalez, Eva Tosco-Herrera, Maria Molina-Molina, Carlos Flores

**Affiliations:** ^1^Unidad de Investigación, Hospital Universitario Nuestra Señora de Candelaria, Santa Cruz de Tenerife, Spain; ^2^Universidad de Santiago de Compostela, Santiago de Compostela, Spain; ^3^Servei de Pneumologia, Laboratori de Pneumologia Experimental, IDIBELL, Barcelona, Spain; ^4^Campus de Bellvitge, Universitat de Barcelona, Barcelona, Spain; ^5^CIBER de Enfermedades Respiratorias, Instituto de Salud Carlos III, Madrid, Spain; ^6^Genomics Division, Instituto Tecnológico y de Energías Renovables (ITER), Santa Cruz de Tenerife, Spain; ^7^Facultad de Ciencias de la Salud, Universidad Fernando Pessoa Canarias, Las Palmas de Gran Canaria, Spain

**Keywords:** Idiopathic pulmonary fibrosis, genetic testing, precision medicine, exome sequencing, telomere length

## Abstract

Idiopathic pulmonary fibrosis (IPF) is a chronic, rare progressive lung disease, characterized by lung scarring and the irreversible loss of lung function. Two anti-fibrotic drugs, nintedanib and pirfenidone, have been demonstrated to slow down disease progression, although IPF mortality remains a challenge and the patients die after a few years from diagnosis. Rare pathogenic variants in genes that are involved in the surfactant metabolism and telomere maintenance, among others, have a high penetrance and tend to co-segregate with the disease in families. Common recurrent variants in the population with modest effect sizes have been also associated with the disease risk and progression. Genome-wide association studies (GWAS) support at least 23 genetic risk loci, linking the disease pathogenesis with unexpected molecular pathways including cellular adhesion and signaling, wound healing, barrier function, airway clearance, and innate immunity and host defense, besides the surfactant metabolism and telomere biology. As the cost of high-throughput genomic technologies continuously decreases and new technologies and approaches arise, their widespread use by clinicians and researchers is efficiently contributing to a better understanding of the pathogenesis of progressive pulmonary fibrosis. Here we provide an overview of the genetic factors known to be involved in IPF pathogenesis and discuss how they will continue to further advance in this field. We also discuss how genomic technologies could help to further improve IPF diagnosis and prognosis as well as for assessing genetic risk in unaffected relatives. The development and validation of evidence-based guidelines for genetic-based screening of IPF will allow redefining and classifying this disease relying on molecular characteristics and contribute to the implementation of precision medicine approaches.

## 1. Introduction

Idiopathic pulmonary fibrosis (IPF), the most common type of interstitial pneumonia, is a chronic and progressive disease in which healthy tissue in the lung parenchyma is progressively replaced by an altered extracellular matrix leading to the loss of lung function (1, 2). This situation leads to dyspnea and abnormal gas exchange, restrictive physiology, hypoxemia, and finally, respiratory failure (3). Treatment options for IPF are limited, although many drugs targeting different pro-fibrotic pathways are being tested in clinical trials (4). Only two antifibrotic drugs, pirfenidone and nintedanib, have been approved for patient treatment by now. Both drugs reduce the decline in lung function, but none is curative (5, 6). Thus, although these drugs may slightly delay the 2–5 years estimated median survival time from diagnosis, disease lethality remains a challenge (7–9).

IPF often clusters in families where multiple members are affected. The term familial pulmonary fibrosis (FPF) is coined when at least two members from the same biological family are affected with IPF (10). In both familial and sporadic forms, common and rare genetic variants contribute to the disease architecture (11). Despite the genetic factors involved in both forms are incompletely understood, genetics is only partially overlapping. Rare variants affecting function of genes of the surfactant metabolism and telomere biology are commonly found in FPF cases when using sequencing studies in kindreds. Particularly, most of these deleterious variants affect telomere genes and they are associated with shorter telomere length (TL) (12). For that reason, obtaining TL measures are also recommended during the diagnostic process (11, 13, 14). The role of common genetic variants in IPF has been identified through genome-wide association studies (GWAS) in sporadic cases (15, 16). For now, nearly two dozen of genetic loci have been associated with IPF susceptibility, although the strongest common risk factor described is a single nucleotide polymorphism (SNP) in the promoter of *MUC5B* (17).

Despite the important recent advances in understanding the genetics underlying disease risks, the current IPF guidelines do not recommend the use of genetic testing for diagnosis (18). In this review, we outline the genetic architecture of IPF and discuss the clinical outcomes of specific variants within IPF genes. We also summarize the molecular technologies which could be applied to assist in the diagnosis of the patients and describe the situations in which it should be performed. Finally, we frame this scenario in the context of the fast developments occurring with the genomic technologies and in how they could further contribute to the implementation of precision medicine in IPF patients.

## 2. Genetics of IPF

**Figure 1:**
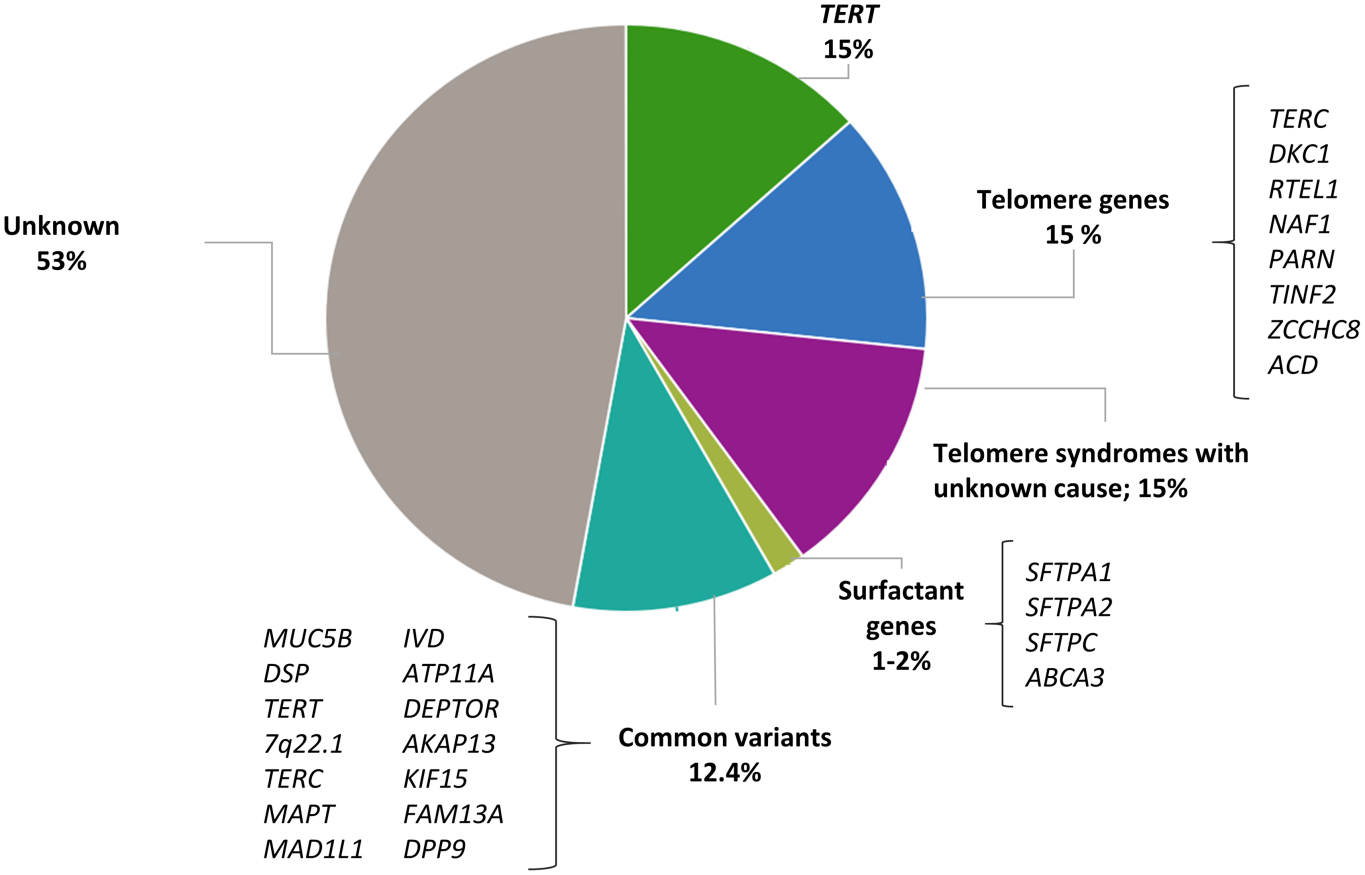
Contribution of rare and common variants to IPF risk. Proportion of risk explained by rare variants in telomere genes and surfactant genes; proportion of patients with short telomere length where a mutation has not been identified; proportion of risk explained by 14 IPF susceptibility variants estimated by Leavy et al. (20).

Genetic factors play a significant role in the development of IPF. The known genetic variants associated with IPF are classified into two broad categories: common SNPs that are broadly found in the general population (allele frequency > 1%), and rare damaging variants in the spectrum of allele frequency below <1% which are typically not recurrent in the general population (19). Next-generation sequencing (NGS) technologies, where the order of nucleotides of the entire genome or of regions of interest are determined, are commonly applied to assess the contribution of rare variants to human phenotypes and have led to the identification of genes that are causal or associated with the risk of the disease. In IPF, most research and clinical sequencing studies have been performed in FPF patients and have identified rare variants in two distinct biological pathways: the surfactant metabolism and telomere maintenance (Figure 1). Collectively, they are found in about 25% of all patients with FPF and, in most of these cases, the disease displays an autosomal dominant (AD) inheritance pattern with incomplete penetrance. Since these rare variants only account for a small proportion of the population attributable risk of IPF, other approaches should be considered to define the role of common variants in the disease. In contrast to rare variants, common variants are present at a higher frequency in the general population although they individually associate with a smaller effect size (reduced penetrance). In GWAS studies, hundreds of thousands of common genetic variants are tested to find those that are statistically associated with a specific trait. If the trait of interest is dichotomous (presence/absence of IPF among the study participants) the study design involves the inclusion of cases (individuals with the trait) and controls (individuals without the trait) (21). This approach has highlighted new genes involved in IPF pathogenesis and possible new therapeutic targets. Thus, taken together, the results emerging from genetic studies support that IPF results from a complex interaction between genetic variants at variable frequency and environmental factors.

### 2.1. The genes involved in IPF development

The genetic studies in IPF have provided significant advances in the understanding of disease pathophysiology. The genes encoding proteins which fall in the same pathway are expected to disrupt the same mechanism and, therefore, can be targeted similarly. This fact reinforces the importance of disclosing the molecular causes underlying disease risk.

#### 2.1.1. Telomere-related genes

Telomeres are specialized structures at the end of chromosomes. In humans, they typically consist of the tandemly repeated TTAGGG nucleotide sequence and the associated protective proteins known as the shelterin complex. Their main functions are to protect genome integrity and prevent degradation and chromosomal end-to-end fusion (22). As the DNA replication machinery is not capable of completely copying DNA at the extreme ends of linear chromosomes, telomeres shorten with each cell division. This situation is resolved in eukaryotes by the telomerase activity, which adds small (telomeric) repeat sequences to the end of chromosomes to compensate this attrition. This complex enzyme involves a catalytic subunit, the telomerase reverse transcriptase (TERT), the telomerase RNA component (TERC), and other relevant components, such as dyskerin (DKC1) and protein regulators (11, 23). However, the activity of the telomerase is limited, and telomeres inevitably shorten throughout the life span. When short telomeres reach a critical threshold across all chromosomes, cellular senescence and apoptosis are triggered by DNA damage responses (24).

Telomere biology disorders are a phenotypic group of diseases that share the molecular defect of short TL due to the presence of germline mutations that affect telomere maintenance (25). Dyskeratosis congenita was the first of these disorders to be described. Affected patients spontaneously develop pulmonary fibrosis at the second decade of life (26). Since then, multiple genes participating in the telomere maintenance have been linked to the pathogenesis of IPF. Gene defects affecting telomerase catalytic enzyme activity (*TERT* and *TERC*) were the first to be described in families with IPF and no history of dyskeratosis congenita (27, 28). With the advent of the NGS technologies, exome sequencing (ES) of patients with FPF have identified at first instance rare variants in two additional genes: *PARN*, which encodes for an enzyme that removes oligo(A) tails from the precursor RNA including that of TERC, and *RTEL1*, which encodes a helicase that unwinds the G-quadruplex and the T-loop secondary structures at the telomeric end (29, 30). Then, case reports have also identified mutations in *DKC1* (31), *ZCCHC8* (32), and *NAF1* (33), all with a role in the biogenesis of telomerase, and *TINF2* (34) and *ACD* (35), implicated in the telomere integrity (Table 1 and Figure 1). Pathogenic rare variants found in these genes lead to alterations in protein function, measured by decreased telomerase activity and telomere shortening in somatic cells, including blood leukocytes, oral mucosal epithelial cells, and lung epithelial cells (27–29, 33, 34, 36–38). Rare pathogenic variants in telomere-related genes are found in approximately one fourth of FPF cases and one tenth of sporadic IPF cases, the variants in *TERT* and *TERC* being the most frequent (8–15%) (27, 28). Rare pathogenic variants in *PARN* and *RTEL1* comprise 5–10% of FPF cases (30). Mutations in other genes such as *TINF2* and *NAF1* collectively represent about 1% of the cases, and they have been identified in a few affected families so far (33, 34) (Figure 1).

**Table 1:** Genes related to IPF and clinical outcomes from causal and associated variants.

Gene family	Gene	Gene function	Variant frequency	Clinical outcomes	References
Telomere biology	*TERT* *TERC* *RTEL1* *DKC1* *ZCCHC8* *NAF1* *PARN* *TINF2* *ACD*	DNA-repair and senescence	Rare and common	Age of lung disease onset: Adult Extra pulmonary symptoms (early onset): dysplastic nails, reticular skin pigmentation, oral leucoplakia, bone marrow failure, liver disease, gastrointestinal disease, premature graying of hair, thrombocytopenia, macrocytosis, immunodeficiency. Rapid progression of the disease Poor survival Hematological complications after lung transplantation Anticipation Short telomere length Familial forms of IPF	(13, 21–44)
Surfactant metabolism	*SFTPA1* *SFTPA2* *SFTPC* *ABCA3*	Lung surfactant and surfactant processing	Rare	Age of lung disease onset: pediatric and adult Familial forms of IPF Lung adenocarcinoma (*SFTPA1* and *SFTPA2*) Respiratory failure in newborns (*ABCA3*) Familial forms of IPF	(45–68)
Susceptibility and prognosis genes	*MUC5B, FAM13A, DSP, OBFC1, ATP11A, DPP9, TOLLIP, MDGA2, SPPL2C, TGFB1, IL1RN, IL8, TLR3, CDKN1A, TP53, ELMOD2, SPDL1, MAPT, MUC2, ZKSCAN1, KIF15, MAD1L1, DEPTOR* *HECTD2, IVD, AKAP13, KANSL1, GPR157, DNAJB4, GIPC2, RAPGEF2, PSKH1, FUT6, MOB2, ACTRT3, ARHGDIG, CRHR1* *GMEB2, PKN2, PCSK6*	*Several*	Common	Sporadic forms IPF *MUC5B* (rs35705950) is related to survival *TOLLIP* (rs5743890) is related to survival *TOLLIP* (rs3750920) is related to efficacious response to oral N-acetylcysteine *PKN2* (rs115982800) associated with forced vital capacity decline *PCSK6* (rs35647788) associated with transplant-free survival	(15–17, 20, 69–81)

IPF, idiopathic pulmonary fibrosis.

Carriers of mutations in telomere-related genes have shortened telomeres, and the phenomenon of genetic anticipation is commonly described in these kindreds. This is defined as an earlier and severe onset of disease associated with the progressive shortening of telomeres of the kindred with each generation. Although approximately 25% of the sporadic IPF patients have telomeres shorter than the 10th percentile of the general population, rare pathogenic variants in genes of the telomere maintenance pathway are only found in 10% of these cases (37, 82). Thus, there are at least 15% of patients with clinical features of short telomere syndrome and short TL where a mutation is yet to be discovered (39) (Figure 1). This fact might be explained by, at least, three main reasons:

1. Additional genes that are critical for telomere maintenance might be involved in the pathophysiology of IPF. These genes may explain smaller fractions of the population risk and could be identified through sequencing additional cases.
2. The short telomeres can be inherited independently of the transmission of the mutations (40). This inherited telomere dysfunction might be sufficient to induce IPF due to persistent DNA damage, inducing cell senescence and alveolar type II cells (AEC2) apoptosis (83, 84).
3. Common variants associated with TL overlap with IPF genes identified through GWAS and might be responsible for the disease in a small fraction of patients where no rare variants were identified (85).

#### 2.1.2. Surfactant-related genes

Pulmonary surfactant is a mixture of lipids and proteins produced by AEC2. Its main function is to reduce surface tension in the alveoli, thereby preventing the collapse of structures at end-expiration. Besides, it is also involved in host defense and the modulation of immune responses. Four surfactant proteins SP-A, SP-B, SP-C, and SP-D are encoded by the *SFTPA1*, *SFTPA2*, *SFTPC*, and *SFTPD* genes, respectively. They are translated within the endoplasmic reticulum (ER) in a pre-protein form and then transported to lamellar bodies where they are stored until they are secreted to the alveolar space. Rare pathogenic variants in *SFTPC* and *SFTPA1-2* have been associated with both adult-onset and pediatric forms of IPF (45, 86) (Table 1 and Figure 1).

The first genetic association between the surfactant and IPF was obtained when Nogee and colleagues (46) reported a coding mutation in *SFTPC* gene in an infant and her mother, both affected with interstitial lung disease (ILD). Since then, numerous studies have identified more than 40 coding and non-coding rare pathogenic mutations in the gene. These mutations are described in pediatric and adult patients, predisposing to a range spectrum of fibrotic lung diseases which segregate in an AD pattern of inheritance (47–49, 87). One of these variants, predicting a Ile73Thr amino acid change, has been described in multiple cohorts, representing approximately 25–35% of the pathogenic alleles (47, 50, 87). Although multiple variants have been reported, there is a plausible common mechanism contributing to disease pathogenesis. Therefore, variants are mostly found in a BRICHOS domain localized in the SP-C pre-protein. Since this domain is critical for proper folding and trafficking within the secretory pathway, its mutations produce an abnormal pre-protein that accumulates within the ER. This results in ER stress and caspase pathway activation which leads to AEC2 injury (51–53). The frequency of rare pathogenic variants in *SFTPC* is low among cases, accounting for 1–2% of FPF patients (54, 55), although a higher prevalence (25%) was described in a Dutch cohort that could be explained by founder effects (56).

Surfactant protein A has two isoforms, SP-A1 and SP-A2, encoded by two adjacent genes, *SFTPA1* and *SFTPA2* (45). Both isoforms share a highly conserved carbohydrate recognition domain wherein all pathogenic heterozygous rare variants for IPF have been found (57, 58). Thus, pathogenic mutations in this domain result in an aberrant protein accumulation in the ER, producing ER stress (59). In contrast to *SFTPC*, which is only expressed in AEC2, *SFTPA* also expresses in Clara cells and submucosal glands from the respiratory airways, having important functions in host defense (60). Pathogenic mutations in *SFTPA1* and *SFTPA2* have been mainly linked to FPF cases and they usually also associate with lung cancer in adult patients (57, 58, 88) (Table 1).

In addition to those encoding the surfactant proteins, other genes encoding proteins involved in surfactant processing have been also involved in IPF pathogenesis. This is the case of the ATP-binding cassette transporter A3 (ABCA3), expressed in AEC2 lamellar bodies where it participates in lipid transportation (61, 62). Early studies reported homozygous or compound heterozygotic *ABCA3* mutations causing fatal distress respiratory syndrome or childhood ILD (63–65) (Table 1). However, other studies describe pathogenic mutations in *ABCA3* in adults with IPF. Moreover, heterozygous *ABCA3* mutations have been described in combination with *SFTPC* mutations in infants with ILD, suggesting that the gene might be acting as a modifier of the disease severity in individuals with *SFTPC* mutations (66, 89).

#### 2.1.3. Common genetic variants and other IPF genes

##### 2.1.3.1. *MUC5B* variant is strongly associated to IPF

The genetic variant that most strongly associate with IPF susceptibility in the GWAS studies conducted to date is a SNP located in the promoter region of the *MUC5B* gene and identified as rs35705950. The variant was identified in 2011 for the first time in a seminal study that relied first on a linkage mapping approach followed by a fine mapping in familial and sporadic IPF cases (17). The same year, the genetic association was confirmed in another study with independent sporadic IPF cases (90). These initial studies firmly supported that the *MUC5B* variant is associated with a strong increase in IPF risk for both familial and sporadic forms. When measured in terms of odds ratios (OR), heterozygous and homozygous of the risk allele (T) of the polymorphism were estimated in as much as 6.8 (95% confidence interval [CI], 3.9–12) and 20.8 (95% CI, 3.8–113.7) for FPF, and 9.0 (95% CI, 6.2–13.1) and 21.8 (95% CI, 5.1–93.5) for sporadic IPF (Table 1).

*MUC5B* encodes mucin 5B, a major component of mucus which is found in various mucosal surfaces, including the lung (91). The promoter variant is in a critical regulatory domain of the gene (92). Consequently, rs35705950 is associated with significantly higher (37.4-fold increase for carriers of the T allele) *MUC5B* expression in lung tissue among unaffected subjects, although the expression is also higher in subjects with IPF (14-fold increase) than in unaffected controls irrespective of genotype (17).

After this seminal study, subsequent studies conducted in non-Hispanic White (NHW) cohorts have replicated the association of the variant with IPF risk (16, 17, 69–71, 93–95). rs35705950 has also proved to be a risk factor for IPF in Mexicans (96) and in Asian populations (97, 98), albeit the *MUC5B* variant is not as common in these populations as in NHW. One important piece of information in this context is that the prevalence of IPF is higher in NHW than in Hispanics or Asians. In contrast, the disease is extremely rare in African populations, where the T allele is also nearly absent.^1^ Although causation cannot be inferred because the presence of the variant alone is insufficient to cause the disease (T allele is also found in 9% of NHW subjects without disease), these observations illustrate an important positive correlation between the frequency of the *MUC5B*-rs35705950 T allele and the prevalence of IPF.

Besides being considered a dominant risk factor for IPF (69, 95), the *MUC5B* variant has been also associated with the presence of interstitial lung abnormalities (99). In addition, this variant associates with increased risk of ILD in patients with rheumatoid arthritis (100), although no associations have been found with asbestosis or sarcoidosis (95).

##### 2.1.3.2. Other common variants associated to IPF

Other common variants have been associated with IPF risk through GWAS, providing further evidence that multiple common genetic variants have an important contribution to disease risk, with an overall risk likely higher than 12.4–17.7% explained by the common variation all together (20) (Figure 1). In the last decade, large-scale international collaborations have been established to combine independent genomic data across thousands of patients and controls to improve the statistical power and detect novel IPF genes. This strategy has allowed the identification of at least 23 independent genetic loci associated with IPF susceptibility and has revealed novel molecular processes involved in IPF pathogenesis including host defense, cell–cell adhesion, DNA repair, airway clearance, innate immunity, profibrotic signaling pathways, mTOR signaling, and mitotic signaling (15, 16, 70, 93, 94, 101).

The first two large GWAS were published in 2013. A GWAS of 1,616 IPF cases and 4,683 control subjects confirmed some previously identified associated loci such as *TERT* and *MUC5B,* and identified novel associations in or near *FAM13A*, *DSP*, *OBFC1*, *ATP11A*, *DPP9*, and in the 7q22 and 15q14-15 regions (70). The same year, a three-stage analysis, comprising 1,410 IPF cases and 2,934 controls in total, also identified associated loci in *TOLLIP* and *SPPL2C* (94). It should be noted that *SPPL2C* is found at 17q21.31. This region includes a well-known pleiotropic inversion polymorphism that is positively selected in Europeans (102). Intriguingly, this locus has been associated with many traits related to lung function such as chronic obstructive pulmonary disease (103), response to inhaled corticosteroids in asthma (104) and, more recently, to severe forms of coronavirus infection (COVID-19) (41). *AKAP13*, was later recognized as another IPF risk gene, therefore the profibrotic signaling pathway represents another potential target in IPF (93) (Table 1).

Recently, two GWAS meta-analyses of IPF have been conducted. The first one was carried out by Allen and colleagues, who confirmed mostly all previously reported signals in addition to identifying three novel signals implicating *DEPTOR*, *KIF15*, and *MAD1L1* genes. These new findings further support the importance of mTOR signaling in IPF. It also suggested cell-cycle progression as another novel biological process, since *KIF15* and *MAD1L1* are both mitotic spindle assembly genes. Moreover, estimates of polygenic risk scores (PRS) for the first time in IPF showed that the disease is highly polygenic and there are potentially hundreds of, as yet unidentified, variants associated with IPF susceptibility (101). The first multi-ancestry meta-analysis of IPF included a total of 8,492 patients and nearly 1.36 million controls from 13 biobanks as part of the Global Biobank Meta-Analysis Initiative. This allowed to identify seven novel loci near *GPR157*, *DNAJB4/GIPC2*, *RAPGEF2*, *FKBP5*, *RP11-286H14.4*, *PSKH1,* and *FUT6* (Table 1). Importantly, only one of these loci would have been associated under the stringent criteria of GWAS studies if the analysis had been restricted only to European populations (16). Thus, the study proves the advantage of increasing sample diversity, as new loci were identified since their index variants were more frequent in other genetic ancestries. The inclusion of understudied populations in GWAS studies is crucial as it allows to fully understand the genetic architecture of IPF while improves the ability to translate the genetic findings into clinical practice independently of ethnicity.

### 2.2. The clinical outcomes and the genetic discoveries

The clinical course of IPF is heterogeneous and yet unpredictable, and the molecular mechanisms underlying the disease are not completely understood. Specific genetic variants have clinical implications in terms of prognosis or associated comorbidities. Therefore, knowing the genetic causes could lead to patient stratification according to the expected prognosis or the treatment response, and contribute to the decision in patient prioritization for lung transplantation (Table 1).

#### 2.2.1. Clinical outcomes of IPF patients with telomere gene mutations

Genetic mutations in telomere-related genes are often linked to the development of a telomeropathy syndrome, which is characterized by a heterogeneous phenotype that includes a wide range of pulmonary and extrapulmonary symptoms. Thus, although ILD seems to be the most predominant phenotype, others such as bone marrow dysfunction, liver cirrhosis, or early hair graying are also described when these mutations are present (23). Importantly, while the prevalence of IPF in carriers of telomere-related mutations increases with age, the extrapulmonary symptoms are often diagnosed at a younger age (105) (Table 1). The age at diagnosis of IPF has been proved to be different also among carriers of mutations: *TERT* mutation carriers are diagnosed at an earlier age (mean of 51 years) relative to mutation carriers in *TERC* (mean of 58 years), *RTEL1* (mean of 60 years), and *PARN* (mean of 65 years) (105).

Clinical lung expression is very heterogeneous among telomerase mutation carriers; chronic hypersensitivity pneumonitis (7–12%), unclassifiable ILD (8–12%), and other idiopathic interstitial pneumonias (14–18%) are described (105, 106). However, the disease evolution is remarkably similar. Among carriers of mutations in *TERT*, *RTEL1*, *PARN,* and *TERC*, one consistent phenotype is progressive deterioration (as measured by absolute forced vital capacity or FVC), suggesting that telomere-related mutations are predictive of disease progression independently of the clinical diagnosis (105) (Table 1).

Telomere shortening has also proven to be a predictor of survival (42), which may condition the stratification of patients with IPF before lung transplantation (43). Furthermore, IPF patients with short telomeres present an increase of specific morbidities after lung transplant such as infection, immunological and hematological dysfunction, potentially impacting on survival (13, 42, 44, 107) (Table 1).

Telomere syndromes are causally linked to the same underlying mechanism, short telomeres, which indicate that telomere-target therapies would be helpful in these patients. These therapeutic interventions are currently being investigated (108, 109). Danazol is a synthetic androgen that preserves TL and improves hematological response in 79% of the patients (110). However, the drug is poorly tolerated, especially due to liver adverse events, and its potential benefit in pulmonary fibrosis is being evaluated in clinical trials (TELO-SCOPE: NCT04638517). Meanwhile, the effect of currently used drugs have been also analyzed in these patients. While pirfenidone and nintedanib seem to be safe and beneficial in IPF patients with telomere related gene mutations and/or telomere shortening (111), a harmful effect of immunosuppression has been suggested (112).

#### 2.2.2. Clinical outcomes of patients with mutations in surfactant biology genes

Surfactant-related gene expression is mainly related to the lung (113), although an increase of frequency in cancer has been also described. In general, the clinical expression related to surfactant genes is variable, but the age of disease onset usually ranges from the infancy to young adulthood (Table 1).

Disease causing *SFTPC* variants have been identified in different ILDs, including idiopathic interstitial pneumonias and pulmonary alveolar proteinosis (46, 47, 49, 87). *ABCA3* gene variants are a common cause of hereditary respiratory failure in newborns (64). These cases are lethal and related to null mutations, leading to the absence of functional ABCA3 protein activity. Instead, milder phenotypes in children or even adults are associated to the absence of null mutations (65, 66, 72, 114). Mutations in *SFTPA2* and *SFTPA1* have been reported only in adults and they are considered a recognized cause of IPF and lung cancer (57). Precisely, this distinction has an impact in transplantation considerations (58).

To date, no specific drug therapy is determined for patients with mutations in surfactant genes.

#### 2.2.3. Clinical outcomes and common gene polymorphisms

The common T allele of the promoter of *MUC5B* (rs35705950), which increases the IPF risk, has been associated with better IPF progression and survival (95, 115–118). Similarly, a G allele in rs5743890 near *TOLLIP,* associated with reduced susceptibility risk, was associated with increased mortality of IPF patients. The latter still lacks replication in independent studies (94).

These data could suggest that carriers of the *MUC5B* promoter variant are part of a subgroup of patients with a distinct prognosis (73, 94, 115). However, these paradoxical findings should be considered with extreme caution. A phenomenon called index event bias, in which a biased association can result from selection of subjects according to their disease status, could explain these results (119). A statistical approximation for adjusting such a bias has been developed and applied to this case. Despite the need for further replication of the findings in independent samples, the results of the application of the novel statistical method suggest that the association of the *MUC5B* variant with survival is biased and the risk allele may, in fact, be associated with decreased IPF survival (119). In this line, the existing evidence based on PRS model inferences suggest that genetic determinants of IPF susceptibility and progression may have limited overlap (101, 120).

Regarding therapies, the PANTHER-IPF clinical trial^2^ was conducted in carriers of the aforementioned *MUC5B* and *TOLLIP* variants to evaluate their modulatory effect on immunosuppressive and antioxidant treatments. The research revealed that, although N-Acetylcysteine did not show efficacy in treating IPF patients in general, a subset of the patients defined by the genotype at rs3750920 could benefit from the drug (121). Independent studies of this assessment are yet lacking from the literature.

Another relevant area of study in IPF involves the identification of genetic variants associated to disease progression rather than disease risk. The first GWAS of decline in lung function in individuals diagnosed with IPF was completed by Allen et al. In this study, the authors discovered a genetic locus that associated with a more rapid decline in the lung capacity, which lies in the RNA antisense gene *PKN2* (101). Consistently, an inhibitor of this gene, fostamatinib, has been already suggested as a potential drug candidate in acute respiratory distress syndrome in patients with severe COVID-19 (122).

## 3. Genetic testing in IPF beyond TL

It is widely recognized that genetic testing could aid in the diagnosis, influence the clinical management, and assist in predicting the prognosis of some patients with IPF. However, there is no consensus recommendation about the implementation of genetic testing or how to identify those IPF patients that may benefit more from this practice (18). In fact, the diagnosis of IPF is still restricted to the identification of a pattern of interstitial pneumonia based on radiological or histological criteria. The American Thoracic Society (ATS), The European Respiratory Society (ERS), The Japanese Respiratory Society (JRS), and Asociación Latinoamericana de Tórax (ALAT) recently updated the clinical practice guidelines for IPF. However, no recommendations were offered regarding when to pursue genetic testing in patients or how to use these results in the clinical practice (18).

**Figure 2:**
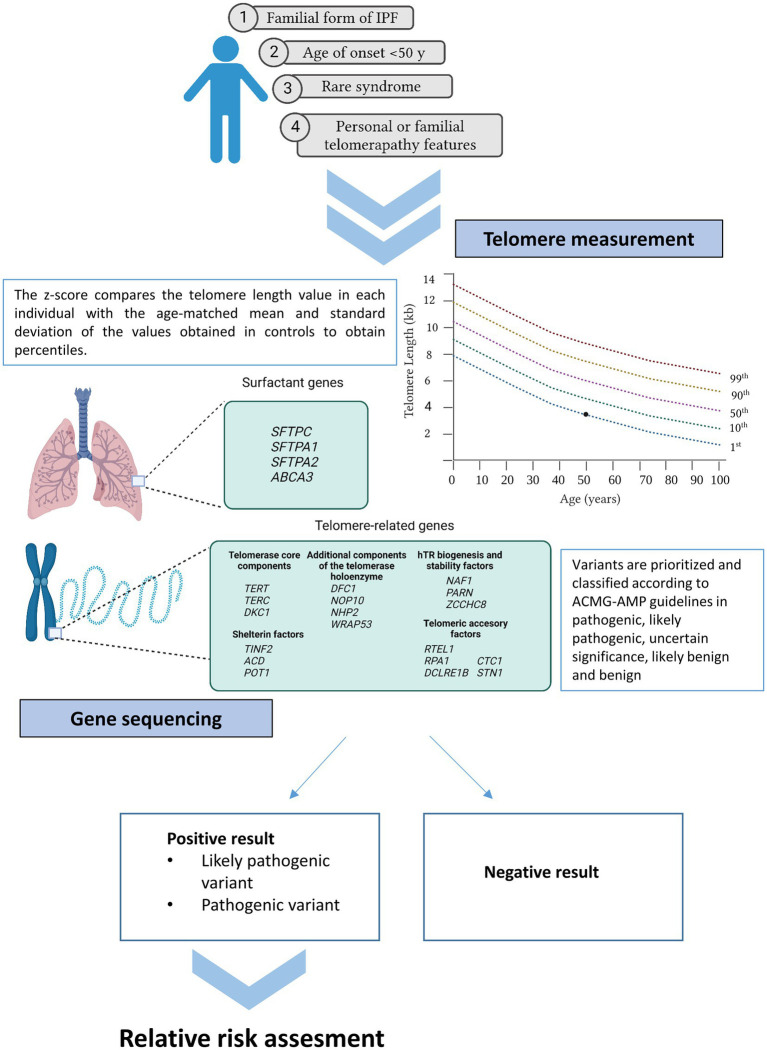
Current recommendations for genetic testing in IPF. Patients meeting at least one of the cited criteria may benefit from genetic testing (telomere length measurement and gene sequencing). If a positive result is obtained in the proband, first relatives can benefit from genetic analysis under request.

Recently, some initiatives developed by pulmonologists, patients, and patient families, confirmed the urgent necessity of guidelines, information, and equal access to patient testing (18, 123). To provide some guidance, the Pulmonary Fibrosis Foundation commissioned a genetic testing group which has reviewed the clinical scenarios in which genetic testing should be offered (125). At the same time, a multidisciplinary expert group of the ERS worked in a statement for better managing FPF, including a question regarding which patients could benefit more from genetic sequencing (11). All of them concur in a series of indications summarized in the following sections (Figure 2).

### 3.1. Methods for clinical genetic testing

The approach for identifying deleterious variants in IPF patients is DNA sequencing. In addition, cases with suspicion of telomere dysfunction can benefit from TL measurement.

#### 3.1.1. TL determination

All methods developed to measure TL face advantages and disadvantages. The most validated methods include qPCR (quantitative Polymerase Chain Reaction) and flow-FISH (Fluorescence *In Situ* Hybridization). TeSLA (Telomere Shortest Length Assay) and TRF (Terminal Restriction Fragment) analyses have shown to be as reliable as other available options (126). More details about these techniques are provided in Table 2.

**Table 2:** Considerations of the available technologies for telomere length (TL) measurement.

Method	Starting material	Measure	Technique	Advantages	Disadvantage	References
TRF	DNA	Average TL length	DNA is digested using restriction enzymes. However, telomeric and subtelomeric sequences remain intact as these sequences are not recognized as digestion sites for the enzymes. After the enzymatic digestion, telomeric fragments are separated based on their size using an agarose gel electrophoresis and are detected by southern blotting and a specific labeled probe for telomeric DNA	Gold standard; specialized equipment not required	Large amount of starting material; labor intensive; extremely short telomeres are difficult to detect; variability between laboratories depending on the restriction enzymes of the experiment	(123, 126-128)
qPCR	DNA	Average TL length	It provides a relative TL (T) compared to a single copy gene (S). The results are expressed in T/S ratio, which is proportional to the average TL. Transformation of T/S ratio to Z-score (by age and gender) allows comparisons of TL between individuals of different ages and results from different cohorts or laboratories.	Small amount of starting material required; high-throughput, easy to perform	High variability within and between samples; extremely short telomeres are difficult to detect, results are not given in kb*	(127)
TeSLA	DNA	Measures the distribution of TL of less than 1 kb* to up 18 kb*	It employs an improved ligation method followed by a PCR approach. The amplified restricted fragments for all chromosomes are then detected using the classic Southern blot analysis with hyper-sensitive digoxigenin labeled probe.	Small amount of starting material required; can detect very short telomeres; automatic quantification of TL using a user-friendly software	Labor intensive, low throughput	(130)
Q-FISH methods	Cells	It measures individual TL in single cells	It determines the TL by measuring the fluorescence intensity after hybridization with a fluorescently labeled nucleic acid telomeric repeat probe. Flow-FISH is an adaptation of the Q-FISH approach which combines flow cytometry with the hybridization of fluorescent probes to suspended cells. This technique is commercially available and CLIA certified. For clinical purposes, this technique has proved to be accurate, reproducible, sensitive, and specific.	Recommended technique for clinical routine; able to detect very short telomeres	Labor intensive; not able to detect telomeric repeats below the threshold because of probe hybridization; possible false positive if the probe also binds to interstitial telomeric sequences	(124, 125, 131)
TelSeq	DNA	Mean TL length in kb	A software to bioinformatically perform estimates of TL from WGS data (BAMs files).	No need for further sample collection and experimental procedures; high correlation with laboratory-based assays	High coverage in needed for reliable measures; bias depending of depth of coverage, sample quality and sequencing platform	(84, 132)

TRF, Terminal Restriction Fragment; qPCR, Quantitative Polymerase Chain Reaction; TeSLA, Telomere Shortest Length Assay; Q-FISH, Quantitative Fluorescence *In Situ* Hybridization; Whole Genome Sequencing, WGS. *kilobases.

Severe reduction is generally denoted when TL is below the 10th percentile of normal controls from the population. Clinical reporting of TL provides information about age-adjusted percentiles and are often represented graphically on telograms (Figure 2).

#### 3.1.2. NGS methods and variant interpretation

##### 3.1.2.1. The use of gene panels and ES

NGS allows sequencing of many genomic regions in a rapid and cost-effective way. Sequencing the whole genome, or a targeted portion of it, has proven to be extraordinarily useful for the identification of molecular causes of genetic diseases. Therefore, its use is widely accepted in the clinical routine (133). Two levels of analysis are commonly performed *via* NGS during the clinical diagnosis of IPF: gene panels and ES.

Gene panels examine the sequence of a curated set of genes associated with the interrogated phenotype and they are considered the first-line test for multiple genetic disorders. By limiting the size of the target sequence, the results usually hold higher sensitivity and specificity for detecting pathogenic mutations (133). Moreover, if only genes with a proven established role in the disease are included, the ability to interpret the findings is greater. In the case of IPF, as in many other diseases, there is yet no consensus about which genes to be included in such a gene panel despite the existence of diverse commercial gene panels for it. IPF is a complex disease and there is still little knowledge about which genes might be involved in its pathogenesis. In addition, as larger cohorts are being sequenced, new disease genes are being discovered that may meet criteria for inclusion in the gene panels (133). This could result in different findings provided by different practitioners depending on the genetic solutions employed. One way to theoretically avoid this issue is by performing ES (133). ES focuses on determining the DNA sequence from nearly all protein coding genes (about 1–2%) and adjacent intronic regions across the genome. This region contains most disease-causing variants identified to date. Thus, one of the major advantages of this technique is the possibility to filter ES data and restrict the analysis to those genes specifically related to the disease. This approximation is equivalent to a virtual gene panel and provides the flexibility to expand the analysis by re-visiting the pre-existing ES data if new disease genes are identified, as it might be the case for IPF (134, 135).

##### 3.1.2.2. Variant calling, filtering, and the challenge of variant prioritization

**Figure 3:**
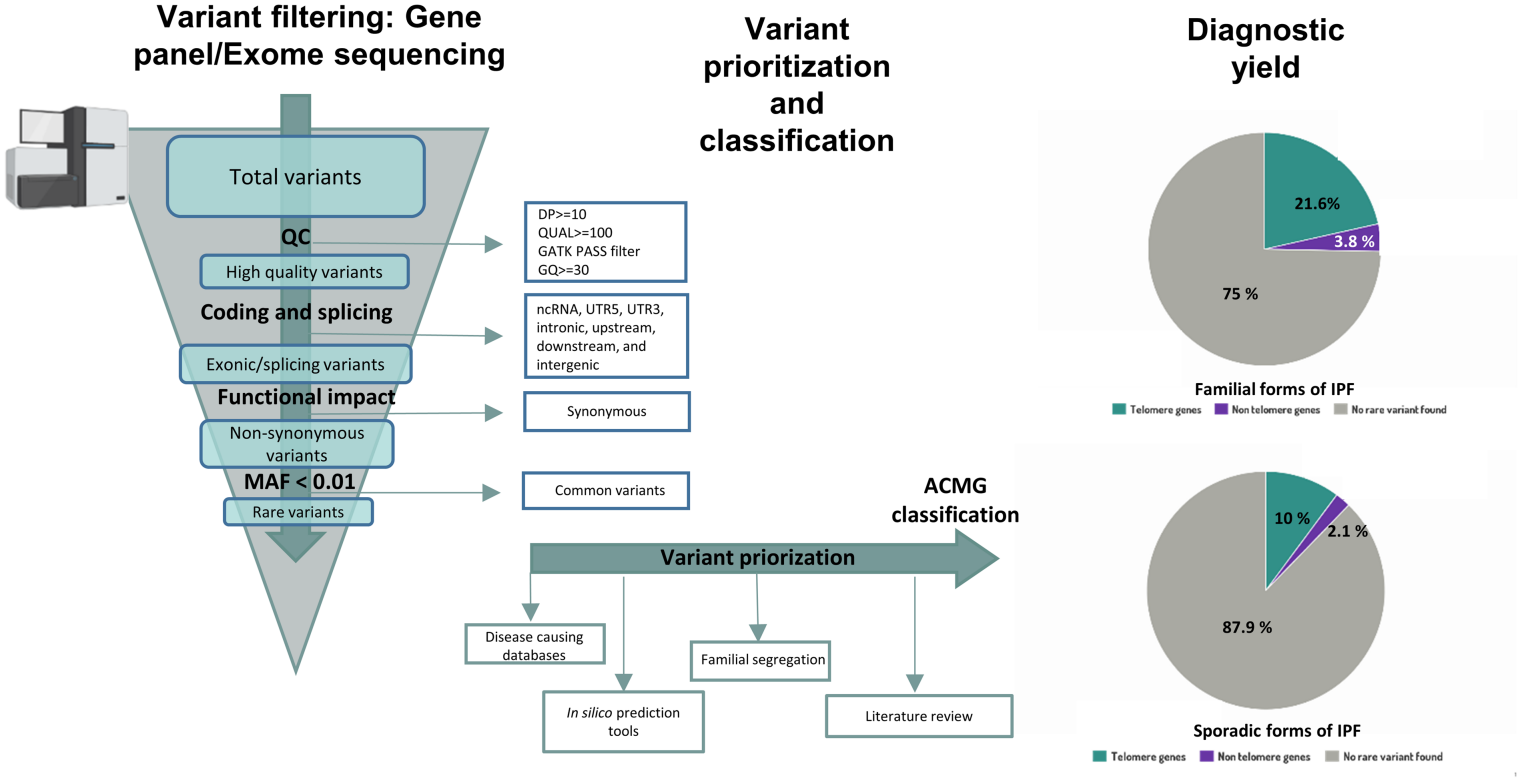
Workflow for variant interpretation in IPF. Annotated variants are filtered applying quality control metrics. High quality coding variants are filtered by predicted consequences on the protein level and non-coding RNA variants, intronic variants and variants inside 5’ or 3’ UTR are discarded. Population allele frequency filters are used to restrict following analysis of rare variants. For variant interpretation, disease causing databases, in-silico prediction tools, literature review and information about familial segregation can be used. Diagnostic yield in familial and sporadic forms of IPF from Zhang et al (12) is shown in pie charts.

Independently of the technology of choice, following the steps of variant calling, filtering, and variant interpretation is critical to provide a precise result (Figure 3). Initially, bioinformatics analysis is required to align the target sequence (gene panel or exome) against the reference sequence of the human genome (136, 137). Thus, patient and reference sequences are compared, and any supported change detected by the variant calling algorithm is further subjected to additional filtering steps and added to the list of variants for the patient. In an ES analysis, however, this process can generate up to 20,000 variants hindering a manual examination (138). For that reason, variants are typically prioritized based on annotated data for relevant biological information such as genomic coordinates, coding sequence nomenclature, protein nomenclature and position relative to the gene. Additionally, relevant information from external resources such as population information or disease specific databases can also be included and used for prioritization and to facilitate the assessment of their clinical relevance (139). One of the first filters to apply uses information regarding the frequency of the variants in the general population from different ethnicities. Although common variants also contribute to the risk of IPF, they are recurrent in the population and their clinical implications are yet unknown. Because of this, genetic testing focuses on identifying rare, non-recurrent, and highly penetrant variants which can be causative of the disease. Therefore, specific thresholds of frequency can be set to restrict the search for rare variants of potential interest. Population databases such as gnomAD^3^ are used with that purpose (139). However, it cannot be assumed that only healthy individuals are represented in these public population databases, especially in the case of a late-onset disease such as IPF. Another useful information to assist in variant interpretation comes from disease-causing databases, for example the Human Gene Mutation Database (140) or ClinVar (141), which contain variants previously observed in affected individuals. Specifically for IPF, The Telomerase Database^4^ compile known mutations that cause human telomerase-deficiency diseases and their associated clinical phenotypes (141). In FPF, in which more than one family member is affected, is also relevant to sequence different family members to assess familial segregation. Thus, if a variant segregates with the phenotype in the family, it constitutes further evidence to support its pathogenicity (139). Finally, the functional consequence of the variant should be considered. *In silico* predictive pathogenicity tools, such as Sorting Intolerant from Tolerant (SIFT), PolyPhen2, Mutation Taster, Variant Effect Predictor (VEP), or the Combined Annotation Dependent Depletion (CADD) score can help in this step, especially for missense mutations (139). All these predictors, however, should be used with caution, as they usually use a fixed cutoff value, identical for all genes in order to filter out benign variants from the NGS data. To improve the use of some of these variant-level methods, the mutation significance cutoff (MSC)^5^ introduced gene-level and gene-specific phenotypic impact cutoff values. Thus, the MSC of a gene is defined as the lower limit of the 99, 95% or 90% CI for the CADD, Polyphen-2 or SIFT scores of all high-quality mutations described as pathogenic in the Human Gene Mutation Database or ClinVar database (142).

After considering the above information, resulting variants are classified at the moment following recommendations from the American College of Medical Genetics and Genomics (ACMG) and the Association for Molecular Pathology (AMP) in five categories: pathogenic, likely pathogenic, uncertain significance, likely benign, or benign (143). A positive result indicates that a patient harbors a pathogenic or likely pathogenic variant in a gene previously implicated with IPF, and therefore, it provides a molecular diagnosis. A negative result, however, means that the causative variant was not identified. Unfortunately, this is the most common result of a genetic testing. However, it should be noted that a negative result does not imply that there is no genetic cause associated to the condition in the patient. Despite efforts from the ACMG to provide criteria for classifying variants, the pieces of evidence are missing or are in conflict with each other in many cases. In these situations, the variant is reported as a variant of uncertain significance (VUS) (143). The advent of NGS technologies implies that more regions of the genome are sequenced, leading to a rapid increase in the number of VUS that can be identified (144). However, there is growing evidence that VUS reclassification is possible when several approaches are used: (1) functional *in vitro* studies at variant level that mimic the mutations and reproduce similar phenotype changes; (2) cumulative evidence from other cases sharing the same variant and phenotype, submitted to public databases; (3) segregation analysis of the variant between affected cases but within the same family; (4) existence of local population-level data to provide a more accurate allele frequency of the variant; and (5) improvement and updates in genetic variant annotation (145–148). Altogether, this evidence can contribute to new conclusions. The more data is collected for a variant, the higher probability will remain to reinterpret the result and refine its classification. For that reason, the reanalysis of NGS data after some time has improved the diagnostic yield in some settings (134, 149–151). Nowadays, 70% of IPF patients who undergo genetic testing have an unidentified genetic cause (Figure 1). However, diagnostic yield should improve as new genomic data is generated.

It should be noted that the workflow described above is specific for single nucleotide variants (SNVs) and indels (short insertions or deletions). Structural variants (SV) are defined as large regions (affecting more than 50 base pairs of sequence) that could involve changes in number of copies of a sequence (deletions, insertions, and duplications), orientation (inversions), or alterations of the chromosomal location (translocations) (152). They have been largely understudied across all diseases because they are more difficult to identify despite having high impact in the disease by altering gene dosage or regulatory elements that modulate gene expression (152). In the last years, many analytical tools for SV detection from NGS data have been developed and incorporated into the routine of clinical laboratories (152). Appropriate filtering strategies should be applied to identify causal SVs. To this aim, characteristics such as size and gene content, quality control metrics, allele frequency, and known association to the patient’s phenotype should be considered to classify and prioritize SVs out from genetic testing (153). However, SNPs and indels constitute the only types of variation that have been tested in relation to IPF for the moment.

### 3.2. IPF cases that most benefit from genetic testing

#### 3.2.1. Patients with family history of IPF

Family history ascertainment is crucial for identifying familial cases of IPF. Therefore, the construction of a pedigree and the collection of the medical history from relatives within at least three generations are essential (125). Importantly, family history should be updated, as it has been described that up to 10% of sporadic forms will subsequently reclassify to familial forms as new relatives are diagnosed during the follow-up (154). The main reason of this procedure is that rare variants are enriched in families with IPF. These variants are expected to confer higher individual risk and tend to be eliminated from the population by purifying selection (155). These types of variants cosegregate with IPF in families as they alone can cause the disease. Consequently, the identification of the genetic cause in familial cases is higher in comparison with sporadic forms, their genetic diagnosis being estimated in 25% (95% [CI]: 21–30%) (12) (Figure 2).

#### 3.2.2. Patients with extra pulmonary symptoms

Different extrapulmonary manifestations may be considered as part of telomere-related syndrome and suggestive of a telomere disorder. Accelerated telomere shortening manifests in a broad spectrum of symptoms involving tissues with high proliferation rates, such as epithelium or the hematological system (25). IPF patients with liver cirrhosis, bone narrow failure, myelodysplastic syndrome, acute myeloid leukemia, and/or premature hair graying present more frequently a pathogenic telomere-related gene mutation (154) (Figure 2).

#### 3.2.3. Patients with early onset of disease

Classically, IPF is an aging disease that usually affects patients older than 60  years (105). Therefore, disease onset is rare before age of 50 and it might be suggestive of the existence of pathogenic mutations in surfactant or telomere related genes (86). Thus, even in sporadic forms of IPF, genetic testing may be useful in those patients in which clinical symptoms develops at a young age (Figure 2).

#### 3.2.4. Monogenic syndromic diseases

Genetic testing should also be considered when there is a suspicion of a genetic syndrome such as Dyskeratosis congenita, Hermansky-Pudlak syndrome, Coats plus syndrome, Høyeraal–Hreidarsson syndrome, and Revesz syndrome (125). In all of them, pulmonary fibrosis is present along with other symptoms affecting multiple organs. Dyskeratosis congenita was the first recognized telomere syndrome, and it is clinically characterized by the triad of abnormal nails, reticular skin pigmentation, and oral leucoplakia. Affected patients are also at risk of experiencing bone marrow failure and cancer predisposition (156). A severe subset of Dyskeratosis congenita is the Høyeraal–Hreidarsson syndrome, which manifests with progressive bone marrow failure, cerebellar hypoplasia, immunodeficiency, and intrauterine growth retardation (156). The second subset of Dyskeratosis congenita is Revetz Syndrome, which also requires being diagnosed with the presence of bilateral exudative retinopathy and central nervous system calcifications. Hermansky-Pudlak syndrome manifests with oculocutaneous albinism and excessive bleeding of variable severity (156). Finally, Coats plus syndrome, a cerebroretinal microangiopathy with calcifications and cyst, is a rare disorder that mainly affects the eyes, brain, bone, and the gastrointestinal system (156) (Figure 2).

### 3.3. The main genes to test

It is expected that approximately up to 30% of the FPF cases are monogenic and that these cases are enriched with rare variants within genes related to two main pathways: surfactant metabolism and telomere biology (11). For that reason, genetic analysis is usually restricted to the genes from these pathways. Among the surfactant genes, *SFTPC, SFTPA1, SFTPA2,* and *ABCA3* are included as consensus in the analysis (Figure 2), despite mutations in these genes explain a small percentage of the total number of cases (1–3%) (Figure 1) (86). Regarding the telomere biology genes, it still less clear which genes should be analyzed. In the past, most of the patients meeting the criteria for genetic testing only had *TERT* or *TERC* genes sequenced since they were the first genes to underlie AD forms of IPF (157, 158). Moreover, mutations in both genes explain up to 15% of the cases (159). Borie et al. and Kropski et al., based on their own experience, proposed the inclusion of at least *TERT, TERC, RTEL1, PARN, NAF1, DKC1,* and *TINF2* genes in the analysis (11, 160). While others have extended the study to all 188 known genes that have been related to hematological disorders (84). More recently, additional telomere biology genes are being identified as causal of IPF *via* ES in affected families. Mutations in these genes are restricted to a few individuals. However, they contribute to better explain the spectrum of the disease causes and, for that reason, they should be also tested, especially in syndromic presentations. This is the case of *NOP10, NHP2, WRAP53, ZCCHC8, ACD, POT1, RPA1, DCLRE1B, CTC1,* and *STN1*. Rare variants in most of these genes are transmitted through various modes of inheritance (X-linked, autosomal recessive, and AD). Biallelic variants are usually related with syndromic forms such Dyskeratosis congenita, Høyeraal–Hreidarsson syndrome, and Coats plus syndrome (25) (Figure 2).

### 3.4. Utility of TL measurements

Telomere shortening is described in up to 50% of the familial forms (161, 162). In patients with telomerase complex mutations, a severe reduction of TL is observed in 80–90% of the cases. For that reason, some authors have proposed to limit gene sequencing to those patients with short telomeres (154), although some telomere related gene carriers and surfactant-related gene mutations would not be genetically diagnosed. Moreover, most of the techniques for telomere measurement offer an average on TL. However, it has been proved that it is not the average but the shortest telomeres of the cells the determinant factor of the responses. It implies that just a few short telomeres of a cell might trigger the DNA damage response (163). In addition, TL effects varies among telomere gene mutations (105).

Some studies suggest that TL testing should be performed in conjunction with gene sequencing, as it may provide information for variant interpretation and assist the clarification of the functional consequences of candidate variants. Alder et al. showed that age-adjusted TL below the 50^th^ percentile has a 100% negative predicted value for clinically relevant mutations in telomere biology genes. Moreover, TL is also predictive of patient prognosis and the approximate timing of disease onset (131). First degree relatives may also benefit from this practice, even when no telomere biology gene mutation has been found, since they are still at risk of developing the disease if telomere shortening is observed in the kindred. In those cases, periodic clinical screening is recommended (40).

### 3.5. Genetic counseling

Briefly, the standard procedure for those patients who meet criteria for being tested is to provide them with relevant information about the method of testing and receive the required educational preparation and training about the benefits and risks of the process (164). In an optimal setting, this information is typically provided by genetic health care professionals (genetic counselors or clinical geneticists) which are members of a multi-disciplinary team (11, 132).

Pre-test counseling in a complex disease, such as IPF, is challenging but also necessary. Understanding the implication of genetic factors in the disease is a crucial step of the process which helps patients to understand the potential results and improve their decision-making. They must be provided with basic concepts of genetic inheritance. In addition, patients who receive genetic counseling should also understand complex concepts such as penetrance or the genetic anticipation (as could be the case for telomere disorders) because of the influence in the natural history of the disease in other family members (127). Thus, patients should understand that the potential results have consequences for them but also for their relatives. Identifying a pathogenic variant may influence the clinical management and may confer prognostic information. Despite this finding should prompt consideration of further genetic testing in asymptomatic relatives, the benefits of this are yet unclear in IPF (see next section). Another challenge arises when a VUS is identified instead of a pathogenic variant. This is, in fact, the most frequent scenario in FPF cases. Patients should be aware of this possibility and understand that VUS can be reclassified as either disease-causing or benign in the future (165). Finally, it should be clear that a negative result does not exclude the presence of a disease-causing variant in IPF genes that remain undiscovered. Thus, the findings of the genetic testing might be provisional results.

The current real situation is that there are wide differences between countries regarding aspects related to genetic counseling such as laws, health systems, or culture (166). Furthermore, in most centers, pulmonologists are responsible for providing information relative to the genetic tests and for communicating the results due to the lack of geneticists or genetic counselors that are integrated to ILD centers (132). A recent international survey revealed the urgent need for improving the genetic counseling in familial ILD by training ILD pulmonologists in genetics and/or including geneticists and genetic counselors in the diagnostic teams (123).

## 4. Future perspectives

In recent years, the application of high-throughput genomic technologies has led to the conclusion that a great inter-individual variation exists regarding the mechanisms that contribute to the disease. This variability is expected to impact its treatment, monitoring and diagnosis. In this review, we have summarized some important genetic aspects of IPF and how the current knowledge is starting to guide the molecular diagnosis of patients and relatives. However, this field is continuously evolving, and new approaches are becoming available and are expected to contribute to the implementation of precision medicine in the healthcare system.

Common and rare variants, as well as TL information can inform about disease risk and outcome of IPF patients. In addition, unexplored types of genetic variants of interest for IPF, such as SVs or variants affecting non-coding regions, are expected to be discovered as larger cohorts are included in genomic research studies (16). Despite the wide spectrum of discovered variants involved in IPF pathogenesis, genetic screening in clinical settings is not yet a common practice for the identification of rare variants with high effect sizes using gene panels or ES. Whole genome sequencing (WGS) has proven to increase the diagnostic yields in other diseases, thanks to the improvement of coding coverage consistency, increased power for detecting SVs, repeat variants, and sequencing of newly annotated coding-regions (167). Particularly in IPF, WGS offers an important advantage over ES due to its ability to identify rare and common variants, and to simultaneously estimate the TL (12). Thus, most rare deleterious qualifying variants in IPF patients are found in telomere biology genes (85%), while non-telomere biology variants are found in a smaller proportion (15%) (12). To determine the biological consequence of these variants, it is necessary to provide TL measures to verify that there is a correlation between the candidate variant effect and the TL. TL, however, is not usually provided by genetic testing laboratories and measures are usually given by alternative services which commonly apply Flow-FISH or qPCR approaches. Telseq and other approaches can bioinformatically estimate TL from WGS data (168) (Table 2). Some of these methods have shown a high correlation with Southern blot and Flow-FISH results (131, 168, 169). For that reason, this approach is becoming more widely used in large cohorts in which a reliable telomere measure is needed (85). However, bias in TL estimation may exist depending on depth of coverage, sample quality, and the sequencing platform used, and all these factors should be taken into account when candidate variants are prioritized (168). A further advantage of WGS is the possibility of detecting common variants beyond gene exons, which also contribute to the risk of IPF. However, effect sizes of individual common variants are very small. For that reason, they are usually discarded for genetic testing. One way to incorporate their information into the analysis is to measure their aggregated effect using PRS (170). The classic way to calculate the PRS is computing the sum of risk alleles that an individual harbors, weighted by the risk allele effect size as estimated by a GWAS on the phenotype (171). If a large number of variants are considered, a substantial greater predictive power is achieved (172). PRS calculated from risk variants previously linked to leukocyte TL (173) has been associated with short TL and disease progression (12). However, the estimation of PRS is not useful for all IPF patients and is not yet a standardized procedure despite its potential. Besides, both familial and sporadic forms might have a different genetic background, meaning that different approaches need to be applied for genetic screening purposes in these two situations. In agreement with this idea, the effect of common variant built PRS is notable only for sporadic cases (those who current guides do not recommend applying genetic screening). Instead, familial forms are five times more likely to be affected by an inherited rare pathogenic variant (12). An updated review of the personal and clinical utility of PRS in complex diseases has been published elsewhere (174).

Another important point of discussion regarding genetic testing is the possibility of offering it to asymptomatic family members. In general, if a monogenic disease is proven in a family, first-grade relatives may benefit from genetic screening if they can make their own informed decision. However, penetrance and expressivity (severity) in IPF is variable and should be carefully considered and explained when the subject receives the related information before being tested (125, 132, 160). The phenomenon of anticipation also complicates the decision. Genetic anticipation is observed under a progressively earlier age of onset and increase of severity of symptoms in each generation. Anticipation is frequently observed in families where IPF is caused by mutations in telomere-related genes and the mechanism that mostly explain it is believed to be telomere shortening (26, 105, 157). It has been suggested that the inheritance of short telomeres in non-mutation carriers of these kindreds might be a sufficient cause to induce the expression of the disease (40). In those cases, it is not the absence of telomerase but rather the short telomeres themselves that cause stem cell failure, as it has been shown in studies conducted in wild type mice with inherited short telomeres (26, 67). The latency before the accumulative effect of telomere shortening leading to IPF is still unknown, although a recent study of the frequently observed pathogenic *TERT* c.2005 T mutation in a Dutch cohort estimates the origin in a founder individual who lived in The Netherlands 300 years ago (68). For all of these reasons, there is no evidence-based practice about when and what tests (DNA sequencing and/or TL measures) should be included in the screening of asymptomatic family members. Further research is needed to assess the predictive power of genetic screening in IPF relatives.

Despite the important advances in the study of IPF genetics in recent years, there are still important caveats regarding molecular mechanisms and the genes involved in the disease. Large GWAS studies and exome or genome sequencing studies have been successful in identifying tens of loci associated with disease risk (15, 16, 82). However, it is expected that new risk genes will emerge when larger cohorts are analyzed, involving sequence data or other omics technologies that could be integrated to improve the predictive power of current studies. For example, an approach that has been used in other complex diseases, such as psychiatric disorders, is leveraging shared genetics between multiple phenotypes for discovering new genes. This is done through multi-trait association mapping (MTAG) studies (74). Another successful approach consists of integrating genetic signals into gene expression data and testing the association between the predicted gene expression and a trait through transcriptome-wide association studies (TWAS). In fact, the integration of both strategies in a cohort of IPF patients led to the discovery of two new candidate genes, *MAFK* and *SMAD2*. Both genes are transcription factors that are related to IPF by regulating target genes which are differentially expressed in specific IPF cell types of affected patients (75).

As new disease mechanisms are being unfolded, new target therapies could be formulated. Until now, the only approved drugs for IPF by the U.S Food and Drug Administration are nintedanib and pirfenidone. Nevertheless, multiple clinical trials are ongoing, and it is expected that target therapies for subsets of patients, defined by a particular genotype, will be developed in line with the principles of precision medicine.

## 5. Conclusion

IPF is a devastating disease with genetic predisposition to disease development and progression, already identified in sub-groups of patients such as those with family aggregation. Significant advances have been made in the field of genetics in the last decade. The ultimate goal of genomic research is to understand the complex genetic architecture of IPF. However, integrating all this knowledge into clinical practice is at reach despite the remaining challenges. In this study, we have attempted to provide detailed information of the known genetic basis of IPF and the potential impact of rare and common variants on disease outcomes. Although the lack of consensus guidelines about when to pursue genetic screening, here we expose current update derived from the perspective of different studies. Future research applying WGS or integrating approaches to combine multiple omics data will further help us to unravel the molecular mechanisms driving pulmonary fibrosis. Finally, advances in new compounds targeting identified gene dysfunctions will provide a better therapeutic approach for these patients.

## Author contributions

AA-G and CF conceptualized the study and wrote the first draft of the manuscript. CF and MM-M obtained the funding. AA-G designed the tables and figures. AA-G, ET-H, MM-M, and CF contributed revising the manuscript. All authors approved the final version.

## Funding

This research was funded by the Instituto de Salud Carlos III (PI20/00876; PMP22/00083) and co-financed by the European Regional Development Funds, “A way of making Europe” from the European Union; the agreement OA17/008 with Instituto Tecnológico y de Energías Renovables (ITER) to strengthen scientific and technological education, training, research, development and innovation in Genomics, Personalized Medicine and Biotechnology; and by Cabildo Insular de Tenerife (CGIEU0000219140). AA-G was supported by Ministerio de Universidades (modality Margarita Salas) and ET-H was supported by fellowship from Agencia Canaria de Investigación, Innovación y Sociedad de la Información del Gobierno de Canarias (TESIS2021010046).

## Conflict of interest

The authors declare that the research was conducted in the absence of any commercial or financial relationships that could be construed as a potential conflict of interest.

## Publisher’s note

All claims expressed in this article are solely those of the authors and do not necessarily represent those of their affiliated organizations, or those of the publisher, the editors and the reviewers. Any product that may be evaluated in this article, or claim that may be made by its manufacturer, is not guaranteed or endorsed by the publisher.

## Footnotes

^1^https://gnomad.broadinstitute.org/variant/11-1241221-G-T?dataset=gnomad_r2_1

^2^https://clinicaltrials.gov: NCT00650091.

^3^https://gnomad.broadinstitute.org

^4^https://telomerase.asu.edu/diseases.html

^5^https://lab.rockefeller.edu/casanova/MSC
